# Real-world Effectiveness of Single-Agent Tivozanib in Patients with Metastatic Clear Cell Renal Cell Carcinoma

**DOI:** 10.15586/jkc.v13i3.472

**Published:** 2026-08-05

**Authors:** Varun Nandakumar, Yeonjung Jo, Georges Gebrael, Zeynep Irem Ozay, Laxmi Upadhyay, Krishnam Goel, Nicolas Sayegh, Ayana Srivastava, Micah Ostrowski, Tanner Hardy, Edwin Lin, Vinay Mathew Thomas, Benjamin Louis Maughan, Haoran Li, Neeraj Agarwal, Umang Swami

**Affiliations:** 1 Department of Internal Medicine, Division of Medical Oncology, Huntsman Cancer Institute, Salt Lake City, UT, USA;; 2 Cancer Biostatistics, Huntsman Cancer Institute, University of Utah, Salt Lake City, UT, USA;; 3 Division of Hematology Oncology, University of Texas Health Science Center San Antonio;; 4 Department of Internal Medicine, Division of Medical Oncology, University of Kansas Cancer Center, Westwood, KS, USA

**Keywords:** metastatic clear cell renal cell carcinoma, overall survival, real-world evidence, time to next treatment, tivozanib

## Abstract

Tivozanib, a selective vascular endothelial growth factor receptor (VEGFR) tyrosine kinase inhibitor, is approved for relapsed or refractory metastatic clear cell renal cell carcinoma (mccRCC). However, real-world evidence of its effectiveness is limited. Herein, we sought to assess the real-world outcomes of single-agent tivozanib in patients with mccRCC. This retrospective study utilized the US-based Flatiron Health electronic health record-derived de-identified database. Patients with mccRCC who received single-agent tivozanib were included. Primary endpoints were real-world time to next treatment (rwTTNT) and real-world overall survival (rwOS). rwTTNT was defined as time from start of tivozanib to next therapy or death, and rwOS as time from start of tivozanib to death, with both censored at loss to follow-up. Median rwTTNT and rwOS with 95% confidence intervals (CIs) were estimated using Kaplan–Meier estimator, stratified by the line of therapy. Of the 13,909 patients with renal cell carcinoma in the dataset, 9,732 had clear cell histology. Among these, 145 patients treated with single-agent tivozanib were included in the analysis. The median age was 68 years (IQR, 61–73 years), and 67.6% were males. rwTTNT and rwOS in the third-line therapy setting were 6.9 (95% CI 3.4, 11) and 11.0 (95% CI 8, 21) months, respectively, and in the fourth-line setting were 4.7 (95% CI 3.6, 7.5) and 8.4 (95% CI 7.2, 20) months, respectively. In summary, single-agent tivozanib potentially provides clinically meaningful benefits in heavily pretreated patients with mccRCC. These findings support its role as a useful later-line therapeutic option in mccRCC management.

## Introduction

The therapeutic landscape of metastatic clear cell renal cell carcinoma (mccRCC) has evolved substantially with the introduction of multiple vascular endothelial growth factor receptor tyrosine kinase inhibitors (VEGFR TKIs), immune checkpoint inhibitors (ICIs), combination regimens of VEGFR TKIs plus ICIs and, more recently, hypoxia-inducible factor-2 alpha (HIF-2α) inhibitors (1). ICI-based combination regimens are now the preferred first-line standard of care, after demonstrating superior efficacy over TKI mono-therapy (2). However, management beyond the first or second line remains heterogeneous, with most patients receiving sequential single-agent VEGFR-TKIs based on prior exposure, tolerability, and comorbidities (3).

Tivozanib is an oral, highly selective inhibitor of VEGFR-1, VEGFR-2, and VEGFR-3, with minimal off-target activity compared with other multitargeted TKIs (4). In the pivotal Phase III TIVO-3 trial, tivozanib significantly improved progression-free survival (PFS) when compared with sorafenib in patients with relapsed or refractory mccRCC (5.6 months vs 3.9 months, Hazard ratio (HR): 0.73; 95% Confidence Interval (CI) 0.56–0.94; P = 0.016). These findings led to the US Food and Drug Administration (FDA) approval of tivozanib in March 2021 for adults with relapsed or refractory advanced RCC after two or more prior systemic therapies (5, 6).

While TIVO-3 established the efficacy of tivozanib in a trial-eligible population, evidence describing its effectiveness in routine clinical practice remains limited. Therefore, we evaluated real-world outcomes associated with single-agent tivozanib in patients with mccRCC using a large real-world database.

## Methods

### Data source

This retrospective study utilized the US-based, electronic health record-derived, de-identified Flatiron Health Research Database, which captures comprehensive, longitudinal oncology data from community and academic practices across the nation (7). Approval for this study was granted by the Institutional Review Board at the University of Utah. Informed consent was not required because the data were de-identified.

### Study population

The study included adult patients diagnosed with mccRCC, who received single-agent tivozanib. The data cutoff was October 31, 2024. Patients were required to have documented first-line therapy.

### Outcome measures and statistical analysis

Real-world time to next treatment (rwTTNT) was defined as the time from tivozanib initiation to initiation of the next systemic therapy or death and was censored at the date of last follow-up. Real-world overall survival (rwOS) was defined as the time from tivozanib initiation to death from any cause, with patients censored at the date of last follow-up according to structured activities. rwTTNT and rwOS were assessed based on the line of therapy using Kaplan–Meier survival estimates accompanied by their respective 95% confidence intervals (CIs). Baseline demographic and clinical characteristics were summarized at the time of tivozanib initiation, including age, race or ethnicity, gender, region, socioeconomic status, practice type, insurance type, line of therapy, and International Metastatic Renal Cell Carcinoma Database Consortium (IMDC) risk groups, using frequencies and percentages. All analyses were performed using R version 4.5.1 (8).

## Results

### Patient demographics and baseline characteristics

Of the 13,909 patients with renal cell carcinoma in the data-set, 9,732 had clear cell histology diagnosed between October 6, 2011, and April 6, 2023. Among these, 145 patients who received single-agent tivozanib between May 16, 2021 and September 3, 2024 were eligible and included in the analysis. The median age at tivozanib initiation was 68 years (inter-quartile range [IQR], 61–73). 37.9% (n = 55) of patients were younger than 65 years, 44.1% (n = 64) were 65–75 years, and 17.9% (n = 26) were older than 75 years. Most patients were male (n = 98, 67.6%) and White, non-Hispanic (n = 98, 67.6%). The majority were treated in community practice settings (n = 100, 69%) and had commercial health plans (n = 89, 61.4%). Most patients were classified as intermediate or poor risk according to the IMDC risk stratification system (n = 114, 78.6%). Further baseline characteristics are summarized in Table 1.

**Table 1:** Baseline patient characteristics at the time of first single-agent tivozanib initiation.

Characteristic	N	%
Age, years, Median (Q1, Q3)	68 (61, 73)	
Age category		
< 65	55	37.9
65–75	64	44.1
≥ 75	26	17.9
Race/Ethnicity		
Black, non-Hispanic	9	6.2
Hispanic/Latino	14	9.7
White, non-Hispanic	98	67.6
Other	13	9
Unknown	11	7.6
Gender		
Male	98	67.6
Female	47	32.4
Region		
Northeast	16	11.0
Midwest	18	12.4
South	47	32.4
West	17	11.7
Unknown/Unclassified	47	32.4
Socioeconomic status (SES) quintile		
1 – Lowest	22	15.2
2	22	15.2
3	36	24.8
4	36	24.8
5 – Highest	18	12.4
Unknown	11	7.6
Practice type		
Academic	40	27.6
Community	100	69.0
Unknown	5	3.4
Insurance type		
Commercial health plan	89	61.4
Medicare/Medicaid/Other government	31	21.4
Other	18	12.4
Unknown	7	4.8
Line of the first single-agent tivozanib initiation		
1	3	2.1
2	11	7.6
3	33	22.8
4	42	29.0
5	24	16.6
6	19	13.1
7	11	7.6
8	2	1.4
Year of single-agent tivozanib initiation		
2021	31	21.4
2022	61	42.1
2023	50	34.5
2024	3	2.1
IMDC (International Metastatic RCC Database Consortium) Risk Group		
Favorable Risk	17	11.7
Poor/Intermediate Risk	114	78.6
Unknown	14	9.7

Percentages are based on the total number of patients who received single-agent tivozanib (N = 145). Continuous variables are presented as median (Q1, Q3); categorical variables are summarized as counts and percentages.

### Treatment patterns

Single-agent tivozanib was initiated most frequently in later lines of therapy. The majority received tivozanib as fourth-line therapy (n = 42, 29%), followed by third-line (n = 33, 22.8%), fifth-line (n = 24, 16.6%), and sixth-line therapy (n = 19, 13.1%). Only a small proportion received it as first-line (n = 3, 2.1%), second-line (n = 11, 7.6%) or eighth-line (n = 2, 1.4%) treatment. Tivozanib use was highest in 2022, with 42.1% of patients (n = 61), followed by 34.5% (n = 50) in 2023 and 21.4% (n = 31) in 2021; only 2.1% of patients (n = 3) received it in 2024 (Table 1).

### Real-world outcomes

**Figure 1:**
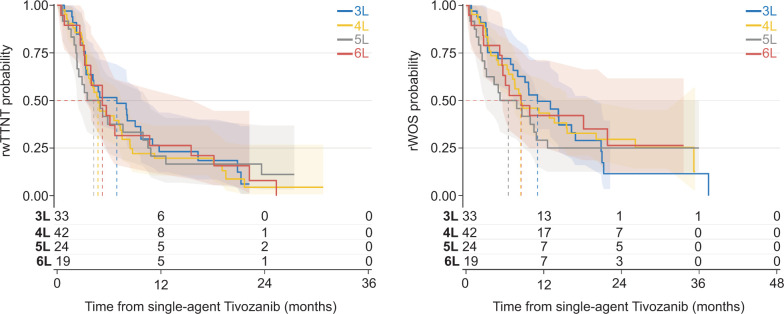
Kaplan–Meier curves showing survival outcomes with tivozanib in patients with mccRCC from the third to the fifth line of therapy. (A) real-world TTNT and (B) real-world OS. Shaded areas represent 95% confidence intervals. Dashed horizontal lines indicate median survival probabilities, and vertical dashed lines denote median times for each group. Numbers at risk are displayed below each panel at selected time points. rwTTNT, real-world time to next treatment; rwOS, real-world overall survival.

Across treatment lines 2–8, the median rwTTNT and rwOS are summarized in Table 2. Median rwTTNT ranged from 6.9 months (95% CI 3.4, 11) in the third line to 4.7 months (95% CI 3.6, 7.5) in the fourth line, 4.2 months (95% CI 2.5, 11) in the fifth line, and 5.2 months (95% CI 3.9, 22) in the sixth line. Consistent with this, the median rwOS was 11.0 (95% CI 8.0, 21), 8.4 (95% CI 7.2, 20), 6.5 (95% CI 3.2, 13), and 8.5 months (95% CI 5.7, not reached (NR)) in the third, fourth, fifth, and sixth lines, respectively (Figure 1).

**Table 2:** Median real-world time to next treatment and real-world overall survival with tivozanib in months and their 95% confidence intervals based on line of therapy.

LOT	N (%)	rwTTNT events	Median rwTTNT in months (95% CI)	rwOS events	Median rwOS in months (95% CI)
2	11 (7.6%)	5	7.4 (3.1, NR)	4	NR (3.1, NR)
3	33 (22.8%)	28	6.9 (3.4, 11.0)	25	11.0 (8.0, 21.0)
4	42 (29%)	37	4.7 (3.6, 7.5)	30	8.4 (7.2, 20.0)
5	24 (16.6%)	21	4.2 (2.5, 11.0)	18	6.5 (3.2, 13.0)
6	19 (13.1%)	18	5.2 (3.9, 22.0)	13	8.5 (5.7, NR)
7	11 (7.6%)	10	3.6 (3.2, NR)	9	12.0 (3.4, NR)
8	2 (1.4%)	2	9.1 (2.9, NR)	0	NR (NR, NR)

LOT, line of therapy; rwOS, real-world overall survival; rwTTNT, real-world time to next treatment; NR, not reached.

## Discussion

In this large retrospective real-world analysis, single-agent tivozanib demonstrated clinical benefit in patients with mccRCC. These findings suggest that tivozanib may be an effective option in routine clinical practice in a heavily pretreated population, supporting its role in patients with relapsed or refractory mccRCC. To our knowledge, this is the largest real-world study to evaluate the effectiveness of tivozanib in a heavily pretreated mccRCC population. Collectively, these results add real-world evidence to the existing clinical data and help inform treatment sequencing decisions in advanced disease.

Only a minority of patients with mccRCC remain eligible to receive later-line therapy due to substantial attrition across treatment lines. Prior analyses have shown that only approximately 27% of patients reach third-line therapy and fewer than 10% proceed to fourth-line treatment, with similar attrition observed in other real-world cohorts (9, 10). Within this context, achieving several months of disease control likely represents meaningful clinical benefit, particularly when therapeutic options are limited.

In the Phase III TIVO-3 trial, tivozanib significantly improved PFS compared to sorafenib but did not show an OS benefit (median OS 16.4 months vs. 19.7 months, HR: 0.99; 95% CI 0.76, 1.29; P = 0.95) (6). The rwOS observed in our third-line and fourth-line cohorts was shorter than that reported in TIVO-3, reflecting differences between trial populations and real-world patients (6). TIVO-3 enrolled patients with Eastern Cooperative Oncology Group (ECOG) performance status 0–1 and applied strict eligibility criteria, which typically enrich for longer survival. In contrast, real-world cohorts include patients with broader comorbidity profiles, variable performance status, heterogeneous prior therapies, and less standardized follow-up.

Real-world studies further highlight the impact of the line of therapy on VEGFR-TKI effectiveness. A UK cohort evaluating first-line single-agent tivozanib reported longer survival outcomes (a median PFS of approximately 8.8 months and OS of 25 months) (11). Conversely, a retrospective analysis of 30 heavily pretreated patients (median of 4 prior lines) reported a median PFS and OS of 3.8 and 14.1 months, respectively, aligning with outcomes observed in our later-line cohorts (12). This gradient of benefit is consistent with prospective trial data. The Phase 3 TIVO-1 trial, which compared tivozanib with sorafenib in the first-line for mccRCC, showed a longer PFS (11.9 months vs 9.1 months, HR: 0.80; 95% CI 0.64–0.99; P = 0.042) and a comparable OS (29.3 months on sorafenib vs 28.8 months on tivozanib, HR: 1.25; 95% CI 0.95–1.62; P = 0.105) with tivozanib (13). Similarly, a retrospective real-world analysis including 64 patients reported favorable response rates and durable disease control with first-line tivozanib (14).

Recent prospective data further inform the role of tivozanib and other VEGFR-TKIs in the post-ICI setting. The phase III TiNivo-2 trial demonstrated no PFS benefit for the combination of tivozanib and nivolumab over tivozanib monotherapy after prior ICI exposure (median PFS, 5.7 months vs 7.4 months; HR 1.10; 95% CI 0•84–1•43; P = 0.49) (15). Similarly, the Phase III CONTACT-03 trial showed no survival benefit from adding atezolizumab to cabozantinib following ICI progression, with increased toxicity in the combination arm (16). These data indicate that ICI rechallenge does not confer additional benefit and support VEGFR-TKI monotherapy as an appropriate later-line strategy in mccRCC.

Tivozanib’s highly selective inhibition of VEGFR-1, -2, and -3 likely contributes to its favorable safety and tolerability profile (17). Although adverse events were not captured in our dataset, prior studies have shown low rates of severe toxicity (17). In TIVO-3, grade ≥3 treatment-related adverse events were similar or lower with tivozanib compared to sorafenib, and fewer patients required dose interruptions or reductions (48% vs 63%) (6). Real-world data further support this tolerability profile, with low rates of treatment discontinuation due to toxicity, an important consideration in older populations (11).

Our study is subject to some limitations. These include retrospective and real-world design, reliance on electronic health records, data missingness, and incomplete outcome ascertainment. Smaller subgroups, such as those in the first-and eighth-line settings, had limited sample sizes and events, which may affect the stability of median estimates. Deaths or off-record treatments may be underreported in the database, and rwTTNT, used here as a proxy for PFS, can be influenced by practice variability and censoring. Additionally, because later-line cohorts represent patients who remained fit and survived long enough to receive multiple lines, our results may overestimate effectiveness compared to an intent-to-treat population. Despite these limitations, this analysis provides important real-world evidence describing single-agent tivozanib outcomes in a heavily pretreated mccRCC population that is underrepresented in clinical trials.

## Conclusion

In this real-world analysis, single-agent tivozanib demonstrated meaningful clinical activity in patients with mccRCC across multiple lines of therapy. The observed effectiveness was generally aligned with the outcomes reported in the Phase 3 TIVO-3 clinical trial, supporting the relevance of tivozanib use outside the clinical trial setting. These findings suggest that tivozanib represents a reasonable treatment option in patients with relapsed or refractory mccRCC. Prospective studies and comparative real-world analyses are required to further define its optimal placement in contemporary treatment sequencing.

## Data Access Statement

The data that support the findings of this study were originated by and are the property of Flatiron Health, Inc. Requests for data sharing by license or by permission for the specific purpose of replicating results in this manuscript can be submitted to PublicationsDataAccess@flatiron.com.

## Mandatory AI Declaration

The authors confirm that no generative AI tools were used in the preparation of this manuscript.

## Author Contributions

Varun Nandakumar: Conception and design; drafting of the manuscript. Yeonjung Jo: Data work; manuscript revision. Georges Gebrael: Drafting and revision. Zeynep Irem Ozay: Drafting and revision. Laxmi Upadhyay: Drafting and revision. Krishnam Goel: Drafting and revision. Nicolas Sayegh: Drafting and revision. Ayana Srivastava: Drafting and revision. Micah Ostrowski: Drafting and revision. Tanner Hardy: Drafting and revision. Edwin Lin: Drafting and revision. Vinay Mathew Thomas: Drafting and revision. Benjamin L. Maughan: Drafting and revision. Haoran Li: Drafting and revision. Neeraj Agarwal: Conceptualization; supervisory role; critical review. Umang Swami: Conceptualization; supervisory role; critical review.
